# Correlates of Zero-Dose Vaccination Status among Children Aged 12–59 Months in Sub-Saharan Africa: A Multilevel Analysis of Individual and Contextual Factors

**DOI:** 10.3390/vaccines10071052

**Published:** 2022-06-30

**Authors:** Chamberline E. Ozigbu, Bankole Olatosi, Zhenlong Li, James W. Hardin, Nicole L. Hair

**Affiliations:** 1 Department of Health Services Policy and Management, Arnold School of Public Health, University of South Carolina, Columbia, SC 29208, USA; cozigbu@email.sc.edu (C.E.O.); olatosi@mailbox.sc.edu (B.O.); jhardin@mailbox.sc.edu (J.W.H.); 2 South Carolina SmartState Center for Healthcare Quality, Arnold School of Public Health, University of South Carolina, Columbia, SC 29208, USA; zhenlong@mailbox.sc.edu; 3 Big Data Health Science Center (BDHSC), University of South Carolina, Columbia, SC 29208, USA; 4 Geoinformation and Big Data Research Laboratory, University of South Carolina, Columbia, SC 29208, USA; 5 Department of Epidemiology and Biostatistics, Arnold School of Public Health, University of South Carolina, Columbia, SC 29208, USA

**Keywords:** infectious diseases, vaccination, immunization, zero-dose, children, sub-Saharan Africa

## Abstract

Despite ongoing efforts to improve childhood vaccination coverage, including in hard-to-reach and hard-to-vaccinate communities, many children in sub-Saharan Africa (SSA) remain unvaccinated. Considering recent goals set by the Immunization Agenda 2030 (IA2030), including reducing the number of zero-dose children by half, research that goes beyond coverage to identify populations and groups at greater risk of being unvaccinated is urgently needed. This is a pooled cross-sectional study of individual- and country-level data obtained from Demographic and Health Surveys Program and two open data repositories. The sample includes 43,131 children aged 12–59 months sampled between 2010 and 2020 in 33 SSA countries. Associations of zero-dose status with individual and contextual factors were assessed using multilevel logistic regression. 16.5% of children had not received any vaccines. Individual level factors associated lower odds of zero-dose status included mother’s primary school or high school education, employment, use of antenatal care services and household wealth. Compared to children in countries with lower GDP, children in countries with relatively high GDP had nearly four times greater odds of being unvaccinated. Both individual and contextual factors are correlated with zero-dose status in SSA. Our results can inform efforts to identify and reach children who have not received any vaccines.

## 1. Introduction

Vaccine discovery and mass distribution through the Expanded Program on Immunization (EPI) established in 1974, remains one of the greatest public health achievements in reducing morbidity and mortality among children globally [1,2,3]. Since EPI debut, many under-five children in low-and-middle income countries (LMICs) have been saved from catastrophic diseases such as Polio, Measles, Diphtheria, Tetanus, Pertussis and Tuberculosis [4,5,6,7,8]. The use of vaccines prevents over 2.5 million deaths each year in under-five children [9,10]. Despite this progress, 1 in 5 children in SSA lack access to necessary life-saving vaccines [11]. As a result, more than 30 million African children under age 5 are affected by vaccine-preventable diseases and, of those, more than 500,000 die each year [12].

Because of these benefits, governments, and international organizations have developed innovative strategies to improve vaccination coverage and penetration into hard-to-reach areas. These strategies include, Reaching Every District (RED) approach, Global Immunization Vision and Strategy (GIVS), and the Global Vaccine Action Plan (GVAP) [13,14,15]. Apart from these strategies, over USD 112 billion funding was provided for vaccine coverage between 2000 and 2017 [16]. However, the focus on “coverage” misses a potentially important population: children with zero-dose vaccination status.

World Health Organization (WHO) and United Nations Children’s Fund (UNICEF) estimates show that 14 million and 17 million zero-dose children exist between 2019 and 2020, respectively, and they mainly reside in 10 lower middle-income countries (LMICs) [17,18,19]. This report indicates that the number of children who should have received the required immunization during the eligibility period (i.e., before their first birthday) and the formative years that follow, is growing [17,18,19]. Inability to reach the zero-dose children could be a pointer to why achieving the 90% goal of vaccine coverage at country and regional level remains a hurdle in SSA to date [20].

To ensure that no child is left behind, the World Health Assembly set an ambitious target of reducing the number of zero-dose children by 50% through the Immunization Agenda 2030 (IA2030) [21]. This is also embodied in the Sustainable Development Goals (SDGs) target 3.2, aimed at ending preventable deaths of newborns and under-five children by 2030 [22]. Achieving the goals outlined in both IA2030 and SDGs requires sufficient research and attention particularly directed to the zero-dose children.

Children with zero-dose vaccination status are at increased risk for infections with vaccine preventable diseases (VPDs) such as measles, polio, and pneumonia, during the first 5 years of life [23]. They are also part of the world’s most vulnerable population, more likely to suffer from disability and mortality that could easily be prevented through immunization [24,25,26]. These children are predominantly less privileged, live in rural households, and raised by mothers with little or no education, indicating disparity within countries [27,28,29]. Lack of attention to the zero-dose population may pose a public health threat in the future, leading to re-emergence of eradicated diseases such as polio, and/or potential disease outbreak such as measles.

While many studies have enumerated factors that impact vaccination coverage, uptake, timeliness, and drop-out rates among specific or different immunization in SSA; how these factors affect the zero-dose population have not been comprehensively examined since most studies are country, facility or intervention-specific, limiting their generalizability [4,5,7,8,9]. For example, while a recent study examined correlates of never-vaccinated children in Nigeria, [23] it is not clear how the results could be generalized in other African countries given the vastly different context across countries in SSA. Similarly, a multi-country study shows that unvaccinated children remain undetected and/or underreported by routine monitoring [30]. This study broadly reported on LMICs without accounting for nuances in Africa. A comprehensive multinational study with primary focus in SSA is needed to fill these gaps. Arambepola et al., reported that incomplete knowledge on factors influencing zero-dose children in SSA needs to be properly investigated [25]. Given the growing concern of changing the status of children from zero-dose to fully vaccinated is yet to be addressed, more studies are needed to identify factors affecting zero-dose children [24,31].

The objective of this study is to identify populations and groups of children at greater risk of being unvaccinated (zero-dose). Considering the high under-five mortality rate in SSA and reports that immunization coverage has plateaued in recent years, we hypothesized that the prevalence of children with zero-dose vaccination status would be high. We also hypothesized that both individual and contextual factors play a role in whether or not a child has been vaccinated. In testing this hypothesis, this study aims to define the prevalence of children with zero-dose vaccination status and examine the independent associations of individual and contextual factors with zero-dose vaccination status among children aged 12–59 months in SSA. Our results can inform efforts to identify and reach children who have not received any vaccines. 

## 2. Materials and Methods

### 2.1. Data Sources

We obtained individual and household-level data from the Demographic and Health Survey (DHS) program [32]. The DHS program started over 35 years ago, with over 400 surveys conducted in over 90 countries since inception [33,34]. The DHS data are nationally representative, cross-sectional, household surveys typically conducted every 5 years to track progress related to national development targets at various levels [34]. The DHS surveys are conducted using standard approach designed to allow for comparison between countries or regions [33]. Detailed information is collected on diverse areas including childhood immunization, infant and child mortality, maternal and child health, reproductive health, Malaria, HIV/AIDS and family planning among others [35]. The DHS program uses a stratified two-stage cluster probabilistic sampling design, with response rates typically exceeding 90% [36]. The first stage involves selection of the primary sampling units (PSUs) called the Enumeration Areas (EA) drawn from census files with the probability of selecting a unit proportional to its size within each stratum [33]. In the second stage, a sample of household is drawn from an updated list of households by equal probability systematic sampling in each EA selected [33]. Details about the DHS program and sampling design has been published elsewhere [35]. We selected countries in SSA with a standard DHS conducted between 2010–2020 for the study (i.e., survey conducted within the last 10 years). This yielded a total of 33 eligible countries (Table 1).

We also obtained country-level data from reports published by the World bank: GDP per capita, literacy rate, fertility rate, health expenditure, unemployment rate, physician density, [37] and the Institute for Economics and Peace (IEP): Global Peace Index (GPI), [38] using each country’s most recent available data consistent or closely related to the DHS survey year.

### 2.2. Study Design and Sample Size

This study was a secondary data analysis of existing data on childhood immunization and utilized a pooled cross-sectional study design. Approximately 44,800 children whose mothers are within the reproductive age group (15–49 years) were initially sampled. Our analysis sample includes 43,131 children whose mother had no missing information on vaccination status and important demographic characteristics. 

### 2.3. Measures

#### 2.3.1. Outcome Variable

Our outcome variable was an indicator for a child’s zero-dose vaccination status. We created a binary variable that was equal to 1 if a child had not yet received any childhood vaccine indicated for children between the ages of 12–59 months. To assess this outcome, the interviewer asked mothers two questions. First, “can they present child immunization card that contain the vaccination history?” This initial step was taken to minimize recall bias. Mothers who could not present an immunization card were asked the child specific follow-up question–“ever had any vaccination?”.

Children of mothers who could present child immunization card (reflected in the card to have participated in no episode of vaccination) and of mothers who were not in possession of immunization card and who responded “no” to the child specific follow-up question were categorized ‘zero-dose’ and were coded “1”. Mothers who could present child immunization card (reflected in the card to have participated in at least one episode of vaccination) and responded “yes” to the follow-up question were categorized “vaccinated” and were coded “0”.

#### 2.3.2. Explanatory Variables

The selection of explanatory variables was guided by the literature and data availability and were grouped into two categories: individual factors and contextual factors. Contextual factors were further divided into those collected in the DHS surveys and measured at the household level and those collected from other publicly available data sources and measured at the national level.

##### Individual Factors

Individual factors included child’s sex (male [ref group] or female), child’s age measured in months (12–24 [ref group], 24–36, 37–48, or 49–59),birth order (1 [ref group], 2–3, 4–5, or 6+), birth weight (low, normal, high, or not weighed [ref group]),mother’s age measured in years (15–19 [ref group], 20–24, 25–34, 35–39, 40–44, or 45–49), mother’s marital status (single/widowed [ref group] or married/cohabitating), mother’s education (no education [ref group], primary school, incomplete high school, or completed high school), mother’s occupation (unemployed [ref group] or employed), antenatal care utilization (no visit [ref group], <4 visits, ≥4 visits, or not asked), place of delivery (home [ref group], public hospital, private hospital, or other location), and exposure to mass media (no TV/radio [ref group] or TV/radio). 

##### Contextual Factors

Contextual factors collected in the DHS surveys and measured at the household level included place of residence (urban [ref group] or rural), household wealth index quintile (poorest [ref group], poorer, middle, richer, or richest), and religion (Christian [ref group], Muslim, other religion, no religion, or not asked). Contextual factors measured at the national level included GDP per capita, domestic general government health expenditure (as a percentage of general government expenditure), adult female literacy rate, female unemployment rate, fertility rate, physicians per 1000 people, and Global Peace Index (GPI) overall score. The GPI is a composite index measuring the peacefulness of countries. It is made up of 23 quantitative and qualitative indicators each weighted on a scale of 1–5. The lower the score the more peaceful the country [38]. For government health expenditures, literacy rate, unemployment rate, and fertility rate, we categorized countries into two groups (low [ref group] or high) relative to the median value to provide results that were more easily interpretable. For GDP per capita, physician density, and the GPI, we assigned countries to tertiles (low [ref group], moderate, high) to allow for nonlinear effects. We also included indicators for subregions of Africa defined by the United Nations (Western Africa [ref group], Eastern Africa, Middle Africa, or Southern Africa). 

#### 2.3.3. Control Variable

Given the differential timing of DHS surveys across sample countries, we included birth year as a partial control for common trends over the sample period and to adjust for effects of unobserved temporal factors.

### 2.4. Data Analysis

We applied the method suggested by the DHS to de-normalize the weights for each of the countries, [39] using the United Nations population of women between the ages of 15–49 years (i.e., women of reproductive age) corresponding to the year of survey [40]. Weighted descriptive statistics were performed to summarize the data. We examined the association between zero-dose vaccination status and all explanatory variables. Statistical differences across groups were examined using chi-squared test.

A 2-level multilevel logistic regression analysis was performed to account for the hierarchical nature of the data: individual observations (level one) were nested within countries (level two). Five mixed effect models were fitted as follows: Model 0 (null model, no variables); Model 1: (individual factors); Model 2 (contextual factors measured at the household level); Model 3 (contextual factors measured at the national level), and Model 4 (full model, all individual and contextual factors). Model 1–Model 4 also included birth year as a control variable. We evaluated variance inflation factors (VIFs) to assess multicollinearity among the explanatory variables. A value exceeding 10 was used as the cut-off point [41]. No multicollinearity among the explanatory variables were observed (mean VIF = 1.41, range = 1.0–2.54). We reported both fixed effects (measures of association) and random effects (measures of variation). Fixed effects were summarized using adjusted odds ratios (aORs), with associated 95% confidence intervals (CIs), whereas random effects were assessed by the Intra-class correlation (ICC), median odds ratio (MOR), and proportional change in variance (PCV) [42,43,44,45,46]. We used the Bayesian Information Criterion (BIC) and Akaike Information Criterion (AIC) to assess goodness-of-fit of models. The model with the lowest value was considered the best explanatory model. For variables with 2.5% missingness (religion and antenatal care), the missing values were recoded and included as a separate category in the analysis. In alternative specifications, we removed religion and antenatal care from the full model, respectively. However, our results were not sensitive to these modifications and were qualitatively comparable. We further removed both variables concurrently in the full model, and our results remained unchanged. To ensure representativeness of the sample, the sampling weights were applied to the models. A *p*-value less than 0.05 was deemed statistically significant. All statistical analyses were performed using Stata Statistical Software, version 15.1, College Station, TX, USA StataCorp LLC. 

## 3. Results

### 3.1. Descriptive Statistics and Bivariate Analysis

Table 2 and Table 3 present baseline characteristics and bivariate analyses of individual and contextual factors, respectively, in a weighted sample of 43,131 children aged 12–59 months in SSA. 16.5% of children were zero-dose, i.e., had not received any vaccines. 

#### 3.1.1. Individual Factors 

Among children, roughly one-half were male (50.7%) and one-quarter were older than 36 months (13.2% aged 37–48 months and 12.4% aged 49–59 months). Among mothers, roughly one-quarter were under age 25 (4.4% aged 15–19 years and 21% aged 20–24 years), one-half were between the ages of 25 and 34 (50.5%), and one-quarter were over age 35 (14.9% aged 35–39 years, 7.0% 40–44 years, and 2.2% 45–49 years). Nearly all mothers were married or cohabitating (90.1%), and the vast majority reported no secondary level education (47.6% no formal schooling and 30.8% primary school only). More than 50% of mothers were employed (56.7%). Nearly one-half of children were born at home (48.6%). The second most common place of birth was public hospital (40.3%). More than 60% of mothers reported that their child had not been weighed at birth (60.5%). Compared to children whose birth weight was recorded, children who were not weighed were significantly more likely to be zero-dose (25% versus 9.1%, 5.3%, and 6.0% for low, normal and high birth weight, respectively). While risk of zero-dose status diminished with age, nearly one-tenth of children older than 36 months remained unvaccinated (9.8% and 9.4% of children aged 37–48 months and 49–59 months, respectively). By comparison, 20.1% of children aged 12–24 months and 17.8% of children aged 15–36 months were unvaccinated. Children born to mothers who had no formal education (23.7% versus 12.4% and 5.49% for completed primary and secondary education, respectively), who were unemployed (21.1% versus 13.0%), who did not receive any antenatal care (35.9% versus 13.3% and 8.97% for 1–4 or 4+ visits, respectively) were significantly more likely to be zero-dose. Risk of zero-dose status was strongly associated with place of delivery. Compared to children born in a health facility, children born at home or in other locations were significantly more likely to be unvaccinated (24.6% and 28% versus 7.76% and 6.34% for those born in public and private hospitals, respectively). Compared to those with regular exposure to media, mothers with no TV/radio were significantly more likely to have zero-dose children (20.2% versus 12.7%).

#### 3.1.2. Contextual Factors

A majority of children resided in rural locations (75.0%) and in countries with low GDP per capita (54.7%), low government health expenditure (54.7%), low adult female literacy rate (61.3%), low female unemployment rate (70.8%), and high fertility rate (59.8%). Most resided in countries with a moderate or high overall GPI scores (39.6% moderate and 44.5% high). Nearly one-half of children belonged to households in the lowest two wealth index quintiles (26.3% poorest category and 23.1% poorer category). Over one-half of mothers were Christian (55.4%). Nearly one-third of mothers were Muslim (31.6%). Compared to those residing in urban areas, children in rural areas were much more likely to be zero-dose (19.1% versus 8.67%). Risk of zero-dose status diminished with household wealth. Roughly one-fifth of children in the lowest two wealth index quintiles were unvaccinated (23.5% and 20.3% of children in the poorest and poorer households, respectively). By comparison, 14.8% of children in the middle quintile, 10.4% of children in the richer quintile, and 6.59% of children in the richest quintile were zero-dose. Muslim children were significantly more likely to be zero-dose than Christian children (25.2% versus 12.3%). Compared to children residing in less wealthy countries, children residing in countries with high GDP per capita were more likely to be unvaccinated (20.4% versus 15.2% and 15.3% for countries with low and moderate GDP per capita, respectively). Children residing in countries with a low adult female literacy rate (21.9% versus 7.94%) and in the Western Africa and Eastern Africa subregions (18.2% and 20% versus 11.4% and 8.6% for the Middle Africa and Southern Africa subregions, respectively) were significantly more likely to be zero-dose.

### 3.2. Measures of Association (Fixed Effects)

**Figure 1:**
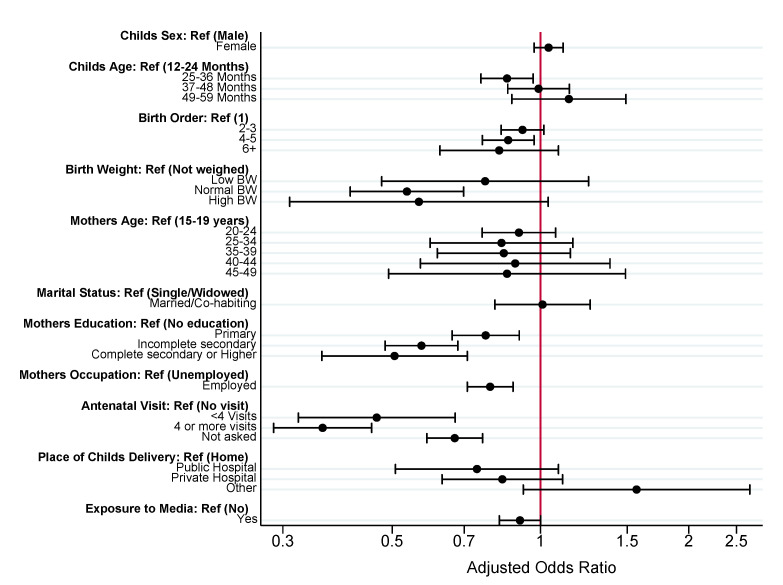
Multi-level analysis of individual factors associated with zero-dose vaccination status among children aged 12–59 months in SSA. Estimates from Model 4 (Table S1). X-axis presented on natural log scale.

**Figure 2:**
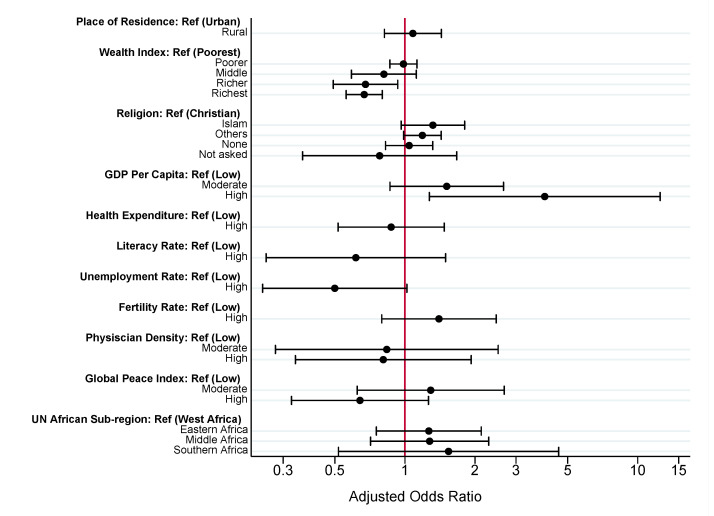
Multi-level analysis of contextual factors associated with zero-dose vaccination status among children aged 12–59 months in SSA. Estimates from Model 4 (Table S1). X-axis presented on natural log scale.

To summarize, in Model 1 (individual factors), child’s age, birth order, birth weight, mother’s education, mother’s occupation, number of antenatal care visits, place of delivery, and exposure to media, were statistically significant predictors of zero-dose status. In Model 2 (contextual factors measured at the household level), rural residence, wealth index quintile, and religion were statistically significant predictors of zero-dose status. In Model 3 (contextual factors measured at the national level), GDP per capita, female unemployment rate, fertility rate and Global Peace Index were the only factors found to be statistically significant. In Model 4 (full model), nearly all individual factors (excluding place of delivery and exposure to mass media) remained statistically significant. Just two contextual factors, household wealth index quintile and national GDP per capita remained statistically significant. Results from Model 4, our preferred specification, are summarized in Figure 1 (individual factors) and Figure 2 (contextual factors) and discussed in greater detail below.

#### 3.2.1. Individual Factors

Children between the ages of 25–36 months were less likely to be zero-dose (aOR: 0.86, 95% CI: 0.76–0.96) compared to children between 12–24 months of age. Compared to first born children, later (fourth or fifth) born children were less likely to be zero-dose (aOR: 0.86, 95% CI: 0.76–0.96). Children with normal birth weight were less likely to be zero-dose (aOR: 0.54, 95% CI: 0.41–0.70) compared to children who were not weighed at birth. The odds of having zero-dose children reduces as mother’s educational attainment increases, such that mothers who completed high school were 49% less likely to have zero-dose children compared to mothers with no education (aOR: 0.51, 95% CI: 0.36–0.71). Similarly, mothers who are employed were 21% less likely to have zero-dose children (aOR: 0.79, 95% CI: 0.71–0.88) compared to mothers who were unemployed. Mothers who received antenatal care were less likely to have zero-dose children, compared to mothers with no antenatal visit, less than four antenatal visits (aOR:0.47, 95% CI 0.32–0.67) and four or more antenatal visits (aOR: 0.36, 95% CI: 0.29–0.45). 

#### 3.2.2. Contextual Factors

Compared to children from the poorest households, children from rich households, children from households in the richer (aOR: 0.68, 95% CI: 0.49–0.93), and richest (aOR: 0.67, 95% CI: 0.56–0.80) wealth index quintiles were less likely to be zero-dose. Countries with high GDP per capita were four times more likely to have zero-dose children compared to countries with low GDP per capita (aOR: 3.99, 95% CI: 1.27–12.48).

### 3.3. Measures of Variation (Random Effects)

As shown in the null model (Model 0, Table S1), there was a significant variation in zero-dose children across the 33 countries (σ2 = 0.79, 95% CI = 0.52–1.20). The ICC coefficient showed low to moderate correlation of zero-dose children within countries (ICC: 19% CI: 14–27%) [47]. The MOR of 2.33 in the null model implied that significant heterogeneity exists across countries. This means that if a child is born in a country with high proportion of zero-dose children, the median risk of being zero-dose would be 2.33 times greater odds. In the full model (Model 4, Table S1), inclusion of the control variable, individual, and contextual factors further reduced the variability within countries (ICC: 5% CI: 4–13%). 

Additionally, the full model showed that unexplained heterogeneity in zero-dose children among the countries decreased from MOR of 2.33 to 1.62. However, there is residual variability of likelihood of being zero-dose at the country level. This implies that if a child is born in a country with high proportion of zero-dose children, the median risk of not being vaccinated would have 1.62 greater odds. Variations in zero-dose children across countries were mostly explained by country-level variables (PCV = 68.35%), followed by individual-level variables (PCV = 34.18%), and household-level variables (PCV = 15.19%).

## 4. Discussion

This multi-country study estimated the prevalence of zero-dose vaccination status and examined the individual and contextual factors associated with zero-dose vaccination status among children aged 12–59 months. Child’s age, birth order, birth weight, mother’s education, mother’s occupation, and number of antenatal care visits were found to be associated with zero-dose vaccination status. Additionally, children from rich households were less likely to be zero-dose than their counterparts from poor households. Countries with high GDP per capita were four times more likely to have zero-dose children compared to countries with low GDP per capita. 

The study findings support our hypothesis of high prevalence of zero-dose children in SSA, with approximately 17% children identified with zero-dose status. To the best of our knowledge, this is the first study to estimate the prevalence of children with zero-dose vaccination status, using nationally representative surveys of 33 countries in SSA. Our study mirrors the result of Bosch-Capblanch et al., who reported prevalence of 9.9% of zero-dose children aged 12–59 months in LMICs [25]. Therefore, the current study underscores the need to increase efforts to move the zero-dose status children to fully vaccinated status in SSA.

Between 1980 and 2010, there was a significant decrease in the number of unimmunized children in SSA, most of which were achieved through the GAVI initiative [48,49,50,51]. In 2019, the Global Burden of Disease (GBD) reported that the majority of zero-dose children are in LMICs [27]. With the IA2030 agenda to vaccinate all children, the Vaccine Alliance launched a global movement to reach these communities and help give zero-dose children a healthy and successful future [51]. To sustain the progress made on early child vaccination, a massive vaccination for all children will be required across the globe, particularly in SSA, where zero-dose children exist the most [26].

Early childhood immunization in SSA is considerably low [52]. Studies have reported that approximately 17 million children in LMIC are yet to receive a single dose vaccines [18,26,28]. The majority of these unvaccinated children live in the inner communities of SSA countries that are difficult to access by healthcare workers [53]. In 2020, as a result of the COVID-19 pandemic, most LMICs faced several challenges in basic healthcare services without exceptions to childhood immunization [25]. Therefore, the number of zero-dose children is expected to double as a result of the COVID-19 pandemic [54]. Apart from COVID-19 pandemic, insurgency, conflicts, and war affects childhood immunization [55,56]. Consequently, these communities are likely to suffer from impending factors such as extreme poverty, gender inequality and lack of social amenities as indicators to influence childhood vaccination coverage. Surprisingly, our study did not find an association between conflict, safety, and peace index of a country to be associated with zero-dose children. 

Other factors that influence zero-dose vaccination include but are not limited to inequality among countries, population size, lack of vaccination services, attitude of healthcare workers toward mothers, maternal education, vaccine hesitancy and political instability [27,31,52,54,57]. Socioeconomic factors as well as perception of the risk of vaccination also pose a huge threat to early childhood vaccination [26,58,59].

The current study further confirms our second hypothesis that both individual and contextual factors play a role in zero-dose children in SSA. For example, mothers who received antenatal care were less likely to have zero-dose children, compared to mothers with no antenatal visit. This holds true in previous studies [60,61,62]. Prior studies have shown that mother’s age and mother’s level of education can influence early child vaccination [52,63,64]. On the other hand, mothers with no education as well as those who are unemployed are less likely to vaccinate their children [65,66,67]. The present study confirmed that mothers with higher educational achievement are less likely to have zero-dose children compared to mothers with no education.

Previous studies have reported that birthweight is an important predictor of childhood vaccination in SSA. O’Leary et al. found that LBW children in Ghana were less likely to receive BCG immunization [68,69]. Their study further indicates that regardless of birthplace, vaccination declines with decreasing birth weight [68]. Similarly, research of Roth et al. conducted in Guinea-Bissau, found that children with LBW were twice as likely to be unvaccinated than those with normal birth weight [70]. The present study confirms that children with normal birthweight were less likely to have zero-dose vaccination status compared to children who were not weighed at birth. Nevertheless, fourth to fifth born children were less likely to have zero-dose vaccination status compared to first born children. This has been reported in a similar study [23].

For more than two decades, scientists, epidemiologists and other stakeholders in the healthcare sector have observed that imbalance in wealth index remains a challenge in delivering healthcare services in SSA [63,71]. Consequently, this study confirms that most households fall within the poorest and poorer categories of wealth index. Households in the middle, richer and richest wealth quintiles are predominantly located in urban areas and likely to have access to good health care facilities. Notably, the wealth index may have a social influence; children from richer households appear to have higher odds of receiving early vaccination than children from poor households, who mainly dominate the zero-dose group. Surprisingly, this study confirms that certain countries with high GDP per capita are four times more likely to have zero-dose children compared to countries with low GDP per capita. This could pertain to the relative wealth index within each country [72]. As such, countries with large revenues from natural resources could appear better off than others even when few people earn sizeable incomes [72]. For instance, Nigeria, with a high GDP per capita, has the highest number of zero-dose children [28]. The number of zero-dose children in countries with high GDP per capita could be attributed to population size, government regulation and unfair health policies.

In light of recent goals set by the Immunization Agenda 2030 (IA2030), including reducing the number of zero-dose children by half, research that goes beyond coverage to identify populations and groups of children at greater risk of being unvaccinated is urgently needed. While much work remains, our results can begin to inform efforts to identify and reach children who have not received any vaccines. Our data suggest that mother’s education, employment, and receipt of antenatal healthcare services may serve as useful indicators in screening tools developed to proactively identify children at risk of being unvaccinated. Screening tools might also incorporate an abbreviated asset index, such as the EquityTool [73], as lower household wealth is strongly associated with zero-dose vaccination status. Our findings also suggest that the number of zero-dose children may be reduced by increasing access to antenatal healthcare services or by educating girls and women. More work is needed to evaluate the effects of specific interventions, including targeted healthcare campaigns and awareness creation. Additional work employing geospatial analysis and modeling strategies could be applied to address issues surrounding zero-dose children and ultimately improve the health and future of children in SSA [25,31,57].

The following limitations of this study are worth noting. First, this study relied on a secondary analysis of publicly available survey data, which might have impacted the findings. Second, survey years and the timing of national measures differ across the countries. To address this, we included birth year as a partial control for common trends over the sample period and to adjust for effects of unobserved temporal factors. Third, given the cross-sectional nature of the data, this study cannot establish causality. However, using nationally representative data from 33 country in SSA, findings from this study is robust and can be used for policy intervention in SSA. It can also help push towards attaining the IA2030 and SDGs agenda. Fourth, to assess the association between zero-dose children and the explanatory variables, the current study utilized multi-level models. Despite the statistical significance of these variables, these models may affect the generalization of the findings presented in our study. Hence, further studies are required to validate the findings presented in the current study.

## Figures and Tables

**Table 1:** Summary of eligible SSA countries and the DHS survey features.

Country	Survey Year	Sample Size (Weighted)
Angola	2015–2016	1458
Burkina Faso	2010	1618
Benin	2017–2018	1403
Burundi	2016–2017	622
Congo DRC	2013–2014	5336
Congo	2011–2012	1882
Cote d’Ivore	2011–2012	767
Cameroun	2018	482
Ethiopia	2016	2407
Gabon	2012	726
Ghana	2014	396
Gambia	2019–2020	101
Guinea	2018	471
Kenya	2014	2219
Comoros	2012	866
Liberia	2019–2020	345
Lesotho	2014	210
Mali	2018	1769
Malawi	2015–2016	515
Mozambique	2011	1703
Nigeria	2018	2670
Niger	2012	2338
Namibia	2013	375
Rwanda	2014–2015	250
Sierra Leone	2019	416
Senegal	2010–2011	1557
Chad	2014–2015	6712
Togo	2013–2014	1013
Tanzania	2015–2016	635
Uganda	2016	557
South Africa	2016	128
Zambia	2018	853
Zimbabwe	2015	331
Total		43,131

**Table 2:** Baseline characteristics and bivariate analysis of individual factors associated with zero-dose vaccination status among children aged 12–59 months in SSA (*N* = 43,131).

Characteristics	Univariate		Bivariate				
	*n*	%	Zero-Dose		Vaccinated		*p*-Value
			*n*	%	*n*	%	
Vaccinated	35,920	83.5					
Zero-dose	7211	16.5					
Child’s sex							
Male	21,684	50.7	3601	16.4	18,083	83.6	0.6898
Female	21,447	49.3	3610	16.7	17,837	83.3	
Child’s age (months)							
12–24	12,884	35.7	2542	20.1	10,342	79.9	<0.0001
25–36	14,836	38.7	2613	17.8	12,223	82.2	
37–48	8034	13.3	1048	9.8	6986	90.2	
49–59	7377	12.4	1008	9.4	6369	90.6	
Birth order							
1	7930	18	1177	15.0	6753	85.0	0.0316
2–3	14,262	32.7	2343	15.9	11,919	84.1	
4–5	10,470	24	1880	16.7	8590	83.3	
6+	10,469	25.4	1811	18.2	8658	81.8	
Birth weight							
Low BW	1733	3.7	140	9.1	1593	91	<0.0001
Normal BW	12,389	26.4	713	5.3	11,676	94.7	
High BW	3,427	8.4	202	6.0	3225	94.0	
Not weighed	25,582	60.5	6156	23.5	19,426	76.5	
Mother’s age (years)							
15–19	2167	4.4	449	21.8	1718	78.2	0.0135
20–24	9254	21	1546	17.7	7708	82.3	
25–34	21,048	50.5	3469	15.6	17,579	84.4	
35–39	6545	14.9	1047	16.1	5498	83.9	
40–44	3073	7.0	502	16.6	2571	83.4	
45–49	1044	2.2	198	17.4	846	82.6	
Mother’s marital status							
Single/Widowed	4722	9.9	586	12.8	4136	87.2	0.0005
Married/Co-habiting	38,409	90.1	6625	16.9	31,784	83.1	
Mother’s educational level							
No education	22,115	47.6	5280	23.7	16,835	76.3	<0.0001
Primary school	12,468	30.8	1351	12.4	11,117	87.6	
Incomplete high school	6204	13.6	450	7.0	5754	93.0	
Completed high school	2344	8.0	130	5.49	2214	94.5	
Mother’s occupation							
Unemployed	18,580	43.3	3982	21.1	14,598	78.9	<0.0001
Employed	24,551	56.7	3229	13.0	21,322	87.0	
Antenatal visit							
No visit	6703	18.1	2750	35.9	3953	64.1	<0.0001
<4 visits	8206	20.1	913	13.3	7293	86.7	
4 or more visits	11,972	28.8	887	8.97	11,085	91.0	
Not asked ^1^	16,250	33	2661	14.5	13,589	85.5	
Place of child’s delivery							
Home	20,522	48.6	5399	24.6	15,123	75.4	<0.0001
Public hospital	18,980	40.3	1323	7.76	17,657	92.2	
Private hospital	2431	7.7	161	6.34	2270	93.7	
Other	1198	3.4	328	28.0	870	72.0	
Exposure to Media							
No TV/Radio	20,497	50.7	4188	20.2	16,309	79.8	<0.0001
Has TV/Radio	22,634	49.3	3023	12.7	19,611	87.3	

^1^ Question not asked in DHS Survey. Questions regarding the number of antenatal care visits were asked in reference to a woman’s most recent pregnancy (youngest child) only.

**Table 3:** Baseline characteristics and bivariate analysis of contextual factors associated with zero-dose vaccination status among children aged 12–59 months in SSA (*N* = 43,131).

Characteristics	Univariate		Bivariate				
	*n*	%	Zero-Dose		Vaccinated		*p*-Value
			*n*	%	*n*	%	
Vaccinated	35,920	83.5					
Zero-dose	7211	16.5					
Place of residence							
Urban	11,223	25.0	1223	8.67	10,000	91.3	<0.0001
Rural	31,908	75.0	5988	19.1	25,920	80.9	
Wealth index							
Poorest	12,902	26.3	2778	23.5	10,124	76.5	<0.0001
Poorer	9430	23.1	1726	20.3	7704	79.7	
Middle	8039	20.0	1270	14.8	6769	85.2	
Richer	7185	17.4	976	10.4	6209	89.6	
Richest	5575	13.2	461	6.59	5114	93.4	
Religion							
Christian	20,339	55.4	2329	12.3	18,010	87.7	<0.0001
Islam	16,181	31.6	3966	25.2	12,215	74.8	
Others	2231	2.8	264	15.0	1967	85.0	
No religion	1222	1.9	240	18.7	982	81.3	
Not asked ^1^	3158	8.3	412	11.1	2746	88.9	
GDP per capita ^2^							
Low	16,540	54.7	2120	15.2	14,420	84.8	<0.0001
Moderate	14,867	20.2	3060	15.3	11,807	84.7	
High	11,724	25.1	2031	20.4	9693	79.6	
Health expenditure ^2^							
Low	28,317	76.2	5361	17.6	22,956	82.4	<0.0001
High	14,814	23.8	1850	13.1	12,964	86.9	
Literacy rate ^2^							
Low	26,057	61.3	5740	21.9	20,317	78.1	<0.0001
High	17,074	38.7	1471	7.94	15,603	92.1	
Unemployment rate ^2^							
Low	27,596	70.8	4753	15.6	22,843	84.4	0.0002
High	15,535	29.2	2458	18.8	13,077	81.2	
Fertility rate ^2^							
Low	17,776	40.2	2492	18.6	15,284	81.4	0.0001
High	25,355	59.8	4719	15.1	20,636	84.9	
Physician density ^2^							
Low	19,315	41.6	3785	20.4	15,530	79.6	<0.0001
Moderate	12,323	31.2	1449	9.51	10,874	90.5	
High	11,493	27.2	1977	18.6	9516	81.4	
Global Peace Index ^3^							
Low	10,765	15.8	1308	13.1	9457	86.9	<0.0001
Moderate	15,777	39.6	2905	21.1	12,872	78.9	
High	16,589	44.5	2998	13.6	13,591	86.4	
UN African Sub-region							
Western Africa	14,864	31.1	2484	18.2	12,380	81.8	<0.0001
Eastern Africa	10,958	35.4	1691	20	9267	80.0	
Middle Africa	16,596	32.2	2997	11.4	13,599	88.6	
Southern Africa	713	1.4	39	8.6	674	91.4	

^1^ Question not asked in DHS Survey. Data unavailable for South Africa, Tanzania, and Niger. ^2^ National measures of GDP per capita, domestic general government health expenditure as a percentage of general government expenditure, adult female literacy rate, female unemployment rate, fertility rate, physicians per 1000 people were obtained from the World Bank [37]. ^3^ National measures of the Global Peace Index (GPI) overall score were obtained from the Institute for Economics and Peace (IEP). The GPI is a composite index measuring the peacefulness of countries. The lower the score the more peaceful the country [38].

## Data Availability

All the analyses were carried out using publicly available datasets that can be obtained directly from the DHS (https://dhsprogram.com/) (accessed 16 April 2022), World Bank (https://www.worldbank.org/) (accessed 16 April 2022), and Vision of Humanity (https://www.visionofhumanity.org/maps/#/) (accessed 16 April 2022) websites.

## Supplementary Materials

The following supporting information can be downloaded at: https://www.mdpi.com/article/10.3390/vaccines10071052/s1, Table S1: Multi-level analysis of factors associated with zero-dose vaccination status among children aged 12–59 months in SSA.
