# Supplementary material for: Correlates of Zero-Dose Vaccination Status among Children Aged 12–59 Months in Sub-Saharan Africa: A Multilevel Analysis of Individual and Contextual Factors
Source: Vaccines (Basel). 2022 Jun 30;10(7):1052. doi: 10.3390/vaccines10071052 (PMC9322920; doi:10.3390/vaccines10071052)
Supplement: Supplementary file 1 [file vaccines-10-01052-s001.zip › vaccines-1784482-supplementary.pdf]

Supplementary Materials: The following supporting information can be downloaded at: https://www.mdpi.com/article/10.3390/vaccines10071052/s1.

Table S1. Multi-level analysis of factors associated with zero-dose vaccination status among children aged 12-59 months in SSA.

	**Model 1**[1]	**Model 2**	**Model 3**	**Model 4**
**Weighted N=43,131**				
**Factors**	**OR (95% CI)**	**OR (95% CI)**	**OR (95% CI)**	**OR (95% CI)**
**Control variable**				
**Birth Year**				
2006[1]	reference	reference	reference	reference
2007	1.45 (0.84-2.49)	1.35 (0.68-2.68)	1.39 (0.66-2.95)	1.41 (0.81-2.47)
2008	2.09 (1.30-3.35)**	1.66 (0.98-2.80)	1.70 (0.96-3.03)	1.99 (1.21-3.30)**
2009	2.11 (1.21-3.68)**	1.57 (0.87-2.82)	1.58 (0.85-2.95)	2.01 (1.11-3.65)*
2010	2.75 (1.48-5.13)**	1.90 (1.04-3.47)*	1.99 (1.03-3.85)*	2.61 (1.34-5.09)**
2011	3.05 (1.51-6.17)**	1.73 (0.94-3.19)	1.82 (0.94-3.51)	2.86 (1.35-6.04)**
2012	3.67 (1.73-7.76)**	1.95 (1.07-3.57)*	2.06 (1.05-4.04)*	3.43 (1.55-7.59)**
2013	4.32 (1.93-9.66)***	2.33 (1.30-4.20)**	2.56 (1.32-4.98)**	4.07 (1.75-9.47)**
2014	4.21 (1.84-9.64)**	2.40 (1.33-4.33)**	2.75 (1.42-5.34)**	3.87 (1.60-9.39)**
2015	3.34 (1.41-7.89)**	2.17 (1.21-3.90)*	2.52 (1.29-4.92)**	3.08 (1.24-7.64)*
2016	4.04 (1.67-9.82)**	2.64 (1.46-4.79)**	3.54 (1.76-7.12)***	3.61 (1.39-9.42)**
2017[1]	3.98 (1.57-10.07)**	2.81 (1.55-5.09)**	4.05 (2.02-8.12)***	3.48 (1.27-9.53)*
**Child's sex**				
Male	reference			reference
Female	1.04 (0.97-1.11)			1.04 (0.97-1.11)
**Child's age**				
12–24 months	reference			reference
25–36 months	0.86 (0.76-0.97)*			0.86 (0.76-0.96)*
37–48 months	1.00 (0.85-1.17)			0.99 (0.86-1.14)
49–59 months	1.15 (0.87-1.52)			1.14 (0.87-1.49)
**Birth order**				
1	reference			reference
2-3	0.93 (0.84-1.02)			0.92 (0.83-1.02)
4-5	0.88 (0.77-0.99)*			0.86 (0.76-0.97)*
6+	0.86 (0.63-1.17)			0.82 (0.63-1.09)

**Birth weight**			
Low BW	0.74 (0.46-1.19)		0.77 (0.48-1.25)
Normal BW	0.50 (0.39-0.64)***		0.54 (0.41-0.70)***
High BW	0.54 (0.30-0.95)*		0.57 (0.31-1.04)
Not weighed	reference		reference
**Mother's age (years)**			
15-19	reference		reference
20-24	0.88 (0.74-1.04)		0.90 (0.76-1.07)
25-34	0.79 (0.53-1.17)		0.83 (0.60-1.16)
35-39	0.79 (0.54-1.14)		0.84 (0.62-1.15)
40-44	0.81 (0.47-1.42)		0.89 (0.57-1.38)
45-49	0.80 (0.43-1.48)		0.86 (0.49-1.49)
**Marital status**			
Single/Widowed	reference		reference
Married/Co-habiting	1.02 (0.81-1.28)		1.01 (0.81-1.26)
**Mother's education**			
No education	reference		reference
Primary school	0.72 (0.59-0.87)**		0.77 (0.66-0.91)**
Incomplete high school	0.49 (0.39-0.60)***		0.57 (0.48-0.68)***
Completed high school	0.39 (0.30-0.50)***		0.51 (0.36-0.71)***
**Mother's occupation**			
Unemployed	reference		reference
Employed	0.77 (0.70-0.85)		0.79 (0.71-0.88)***
**Antenatal visit**			
No visit	reference		reference
<4 visits	0.46 (0.32-0.66)***		0.47 (0.32-0.67)***
4 or more visits	0.35 (0.28-0.45)***		0.36 (0.29-0.45)***
Not asked[2]	0.68 (0.58-0.81)***		0.67 (0.59-0.76)***
**Place of child's delivery**			
Home	reference		reference
Public hospital	0.71 (0.49-1.03)		0.74 (0.51-1.09)
Private hospital	0.74 (0.57-0.96)*		0.84 (0.63-1.11)
Other	1.54 (0.95-2.50)		1.57 (0.92-2.66)
**Exposure to Media**			

No TV/radio	reference			reference
Has TV/radio	0.81 (0.69-0.95)*			0.91 (0.82-1.00)
**Place of residence**				
Urban		reference		reference
Rural		1.52 (1.17-1.97)**		1.08 (0.82-1.43)
**Wealth index**				
Poorest		reference		reference
Poorer		0.85 (0.75-0.96)**		0.99 (0.86-1.13)
Middle		0.60 (0.50-0.73)***		0.81 (0.59-1.12)
Richer		0.44 (0.36-0.55)***		0.68 (0.49-0.93)*
Richest		0.34 (0.20-0.56)***		0.67 (0.56-0.80)***
**Religion**				
Christian		reference		reference
Islam		1.65 (1.28-2.14)***		1.32 (0.96-1.80)
Others		1.45 (1.14-1.84)**		1.19 (0.99-1.43)
No religion		1.23 (0.97-1.57)		1.04 (0.83-1.32)
Not asked[3]		0.89 (0.50-1.59)		0.78 (0.36-1.67)
**GDP per captia[4]**				
Low			reference	reference
Moderate			1.50 (0.89-2.51)	1.51 (0.86-2.65)
High			3.57 (1.36-9.39)*	3.99 (1.27-12.48)*
**Health expenditure[4]**				
Low			reference	reference
High			0.82 (0.45-1.47)	0.87 (0.52-1.47)
**Literacy rate[4]**				
Low			reference	reference
High			0.50 (0.25-1.00)	0.62 (0.25-1.49)
**Unemployment rate[4]**				
Low			reference	reference
High			0.49 (0.28-0.85)*	0.50 (0.24-1.02)
**Fertility rate[4]**				
Low			reference	reference
High			1.68 (1.02-2.76)*	1.40 (0.79-2.47)
**Physician density[4]**				

Low			reference	reference
Moderate			0.51 (0.22-1.18)	0.84 (0.28-2.51)
High			0.71 (0.32-1.56)	0.81 (0.34-1.93)
**Global Peace Index**[5]				
Low			reference	reference
Moderate			1.72 (1.02-2.92)*	1.29 (0.62-2.67)
High			0.90 (0.48-1.66)	0.64 (0.33-1.26)
**UN African Sub-region**				
Western Africa			reference	reference
Eastern Africa			1.39 (0.89-2.17)	1.27 (0.75-2.13)
Middle Africa			1.42 (0.83-2.45)	1.28 (0.71-2.29)
Southern Africa			0.87 (0.44-1.72)	1.54 (0.52-4.57)
**Random effects (country)**				
Variance (95% CI)	0.52 (0.25-1.09)	0.67 (0.38-1.18)	0.25 (0.15-0.42)	0.26 (0.13-50)
ICC (95%CI)[c]	14 (7-25)	17 (10-26)	7 (4-15)	5 (4-13)
AIC[f]	34234	35890	37341	34055
BIC[g]	34512	36080	37566	34332
MOR[d]	1.98	2.18	1.61	1.62
PCV[e]	34.18	15.19	68.35	67.09

Five multilevel logistic regression models were fitted as follows: Model 0 (null model); Model 1: (individual-level variables); Model 2 (contextual factors measured at the household level); Model 3 (contextual factors measured at the national level), and Model 4 (full model). Model 1 – Model 4 also included birth year as a control variable. In Model 0 (null model, not shown), Variance (95%CI) = 0.79 (0.52-1.20); ICC (95%CI) 19 (14-27); AIC=37393; BIC=37410; and MOR=2.33. PCV=ref.

[1] Births in 2005 were recoded to 2006. Births in 2018-2019 were recoded to 2017.
[2] Question not asked in DHS Survey. Questions regarding the number of antenatal care visits were asked in reference to a woman's most recent pregnancy (youngest child) only.
[3] Question not asked in DHS Survey. Data unavailable for South Africa, Tanzania, and Niger.
[4] National measures of GDP per capita, domestic general government health expenditure as a percentage of general government expenditure, adult female literacy rate, female unemployment rate, fertility rate, physicians per 1,000 people were obtained from the World Bank [36].
[5] National measures of the Global Peace Index (GPI) overall score were obtained from the Institute for Economics and Peace (IEP). The GPI is a composite index measuring the peacefulness of countries. The lower the score the more peaceful the country [37].

OR = Odds ratio; CI = Confidence interval; GDP= Gross domestic product; ICC = Intra-class correlation; AIC = Akaike's information criterion; BIC = Bayesian information criterion; MOR = Median odds ratio; PCV = Proportional change in variance.

*p<0.05; **p<0.001; ***p<0.0001.
